# The Effect of Macromolecular Crowding on the Electrostatic Component of Barnase–Barstar Binding: A Computational, Implicit Solvent-Based Study

**DOI:** 10.1371/journal.pone.0098618

**Published:** 2014-06-10

**Authors:** Helena W. Qi, Priyanka Nakka, Connie Chen, Mala L. Radhakrishnan

**Affiliations:** Department of Chemistry, Wellesley College, Wellesley, Massachusetts, United States of America; Instituto de Tecnologica Química e Biológica, UNL, Portugal

## Abstract

Macromolecular crowding within the cell can impact both protein folding and binding. Earlier models of cellular crowding focused on the excluded volume, entropic effect of crowding agents, which generally favors compact protein states. Recently, other effects of crowding have been explored, including enthalpically-related crowder–protein interactions and changes in solvation properties. In this work, we explore the effects of macromolecular crowding on the electrostatic desolvation and solvent-screened interaction components of protein–protein binding. Our simple model enables us to focus exclusively on the electrostatic effects of water depletion on protein binding due to crowding, providing us with the ability to systematically analyze and quantify these potentially intuitive effects. We use the barnase–barstar complex as a model system and randomly placed, uncharged spheres within implicit solvent to model crowding in an aqueous environment. On average, we find that the desolvation free energy penalties incurred by partners upon binding are lowered in a crowded environment and solvent-screened interactions are amplified. At a constant crowder density (fraction of total available volume occupied by crowders), this effect generally increases as the radius of model crowders decreases, but the strength and nature of this trend can depend on the water probe radius used to generate the molecular surface in the continuum model. In general, there is huge variation in desolvation penalties as a function of the random crowder positions. Results with explicit model crowders can be qualitatively similar to those using a lowered “effective” solvent dielectric to account for crowding, although the “best” effective dielectric constant will likely depend on multiple system properties. Taken together, this work systematically demonstrates, quantifies, and analyzes qualitative intuition-based insights into the effects of water depletion due to crowding on the electrostatic component of protein binding, and it provides an initial framework for future analyses.

## Introduction

It is believed that up to 40% of the cellular volume is occupied by macromolecules [1], making the cell a crowded place. Nevertheless, many *in vitro* experiments and computational studies model protein processes in a vast “sea” of aqueous solvent. To build better models of such processes, it is crucial to better understand the effect of cellular crowding on the physical determinants of protein folding and binding. While more attention has been given to these effects in recent years, reviews of crowding effects span multiple decades [2]–[9]. Experimental work has shown that crowding can cause a thermodynamic favoring of compact states – folded, bound, or aggregated states of proteins [10]–[13] – and could favor compaction of unfolded states as well [14], [15], although sometimes certain effects were found to be small or even reversed [16], [17], likely because of enthalpic interactions between crowding agents and the proteins being studied [18]. Nevertheless, even small, subtle effects could have important implications for aggregation associated with neurodegenerative diseases [10], [19]. Crowding has also been experimentally shown to change the preferred conformations of protein and DNA systems [20]–[25] and to alter drug–target interactions or affinities [26]–[28]. Finally, macromolecular crowding may slightly [16], [29] or more greatly affect association rate kinetics [30] and reaction mechanisms [31], [32].

Theoretical and computational studies have provided great insight into the physical bases for observed effects due to macromolecular crowding. Many thermodynamic studies to date have focused on the entropic “excluded volume” effect, in which crowding lowers the available cellular volume, thus lowering the entropy of noncompact states more than that of compact states, leading to a relative free energy stabilization of compact states. This effect was shown to have measurable consequences in theoretical and computational studies [33]–[36]. More recently, it was shown that favorable interactions between less compact states and the crowders could cancel out this effect or dominate over it [37]–[39], demonstrating not only that the physical properties of the crowders are important, but also that crowding could significantly affect the enthalpic component of the binding free energy in addition to the entropic component. The subtle interplay between multiple energetic components as well as dynamical effects have been considered via molecular dynamics simulations of proteins within a crowded environment [37], [38], [40], [41]. These and other time-dependent simulations [42], [43] have also provided insight into the association rates of proteins within the cellular milieu.

There have been relatively few studies that focus on how crowding affects the *electrostatic* component of protein–protein interactions and their solvation energetics. As a reasonable hypothesis, crowding can both affect the hydration dynamics of water [44] and deplete the number of polarizable water molecules surrounding the proteins, thereby potentially descreening their electrostatic interactions relative to the infinite dilution limit (i.e., the uncrowded case). While crowding has been incorporated into electrostatic models via a screened Coulomb potential-based implicit solvent model [45] and a lowered effective solvent dielectric constant [46], to our knowledge, only very recent work has probed more specifically to study how crowding affects electrostatic interactions within a solvated medium [47], [48]. Such work demonstrated that it may be possible to capture certain electrostatic effects of crowding by a lowered solvent dielectric constant, a result that supports other work suggesting that the observed dielectric constants within cellular environments may be quite lower than that of water [49]–[53]. Specifically, Harada *et al*. [47] found via explicit solvent molecular dynamics simulations that water mobility was hindered in a crowded environment, providing one physical mechanism for this lowered dielectric constant. However, as they note, another mechanism for a lowered dielectric constant may stem from the fact that crowding depletes bulk water from around molecules, an idea that was explored further in an implicit model study [48]. It is this latter mechanism that provides the focus of the current study, although here, we extend this idea to study protein–protein binding.

This work uses simplified models to study how water depletion due to crowders can alter electrostatic binding free energies between proteins. We use the barnase–barstar protein complex as a model system, as it has been shown previously [54], [55] that electrostatic interactions play a crucial role in their interaction, and it has also been used in previous studies investigating crowding or similar phenomena [35], [45]. While a more realistic model may use explicit solvent and actual proteins as crowding agents, we wished to separate out electrostatic effects due to water depletion from other electrostatic effects, such as loss of mobility of individual water molecules or electrostatic interactions with crowder molecules. To that end, our study uses spherical, uncharged model crowders within an implicit solvent, and electrostatic free energies are computed through obtaining potentials via the Poisson Equation (or the Linearized Poisson-Boltzmann equation, if applicable). To again focus on the water depletion effect in a controlled manner, we assume rigid binding, although we recognize that crowding may affect protein conformations [48]. Our thermodynamic cycle allows us to separately quantify the effects of crowding on desolvation and on solvent-screened interaction. The use of simple model crowders enables us to systematically study these effects as a function of crowder density and size. Adequately sampling crowder locations to get proper Boltzmann-weighted distributions of states would be computationally infeasible, and so we limited our results to simple averages over 50 randomly-generated crowder placements in the bound and unbound states per data point, especially since Boltzmann-weighting based only on electrostatic solvation energies may be less realistic than assuming that other factors can also contribute to crowder placement.

We find that on average, crowding lowers desolvation penalties and amplifies solvent-screened interactions, stabilizing favorable interactions and destabilizing unfavorable ones. This effect is more pronounced when crowder size is reduced, assuming a standard-size water probe radius within the continuum model. The mean stabilization or destabilization of solvent-screened interactions was robust to the specific placement of the random crowders, but the average desolvation effects were not, with very large standard error values. While an overall reduced dielectric constant may capture average water depletion effects, there may be system specific conditions that lead to uncertainty in the mean effect of crowder placement as a simple function of crowder density and size. Finally, we show that crowding can differentially affect the electrostatic contributions of individual protein residue side chains toward binding, with the relative effects on desolvation and interaction depending on the residue’s environment. This suggests that crowding could affect the consequences of specific mutations on binding, as well as the role that certain residues or binding “hot spots” play in varied cellular environments. While these results may qualitatively agree with intuition, our goal is to provide a systematic, controlled demonstration and quantitative analysis of these effects. Moreover, the methods used here provide experimentally testable hypotheses and an initial framework for understanding the role of crowding in modulating electrostatic interactions in protein–protein binding that can be built upon in future work.

## Materials and Methods

### Structure Preparation

Studies used a 2.0 Å resolution crystal structure of barnase complexed with a Cys -> Ala (40,82) double mutant of barstar (PDB ID 1BRS) [56]. The asymmetric unit consisted of 3 model complexes; the complex corresponding to chains A and D were used in this study. Crystallographic water molecules greater than 3.3 Å from either binding partner or with fewer than three potential hydrogen-bonding interactions with protein were removed. The remaining 17 water molecules were assigned to either protein partner based on proximity and hydrogen-bonding contacts. The amide groups of asparagine and glutamine and the imidazole group of histidine were flipped as necessary and the tautomerization states of histidine were assigned based on manual inspection of possible hydrogen bonding with surrounding residues. The two N-terminal residues of barnase and residues 64 and 65 of barstar were not resolved in the crystallographic experiment, and neighboring residues were patched with acetyl or N-methylamide groups. Hydrogens were modeled onto the structure with the HBUILD [57] functionality in CHARMM [58], using the CHARMM22 force field [59] and the TIP3P water model [60]. Patches and missing side chain density were added via CHARMM and were energy minimized.

### Crowder Placement

**Figure 1:**
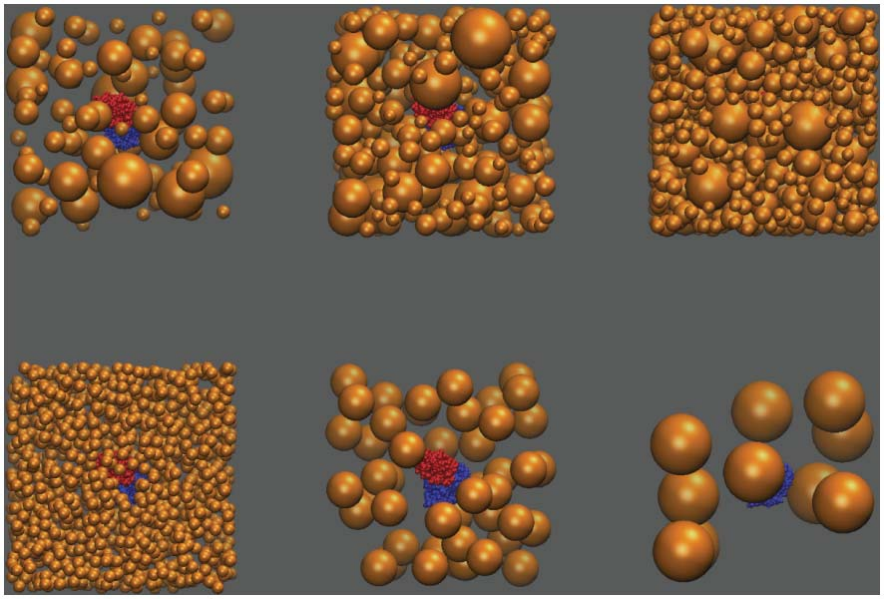
Sample simulated crowded environments. Here, the bound state barnase–barstar complex (red and blue) is surrounded by randomly-placed crowders (orange); the top row depicts environments in which the radius of crowders varied within a system (from 5–25 Å), at increasing crowder volume densities (left to right). The bottom row depicts environments at a constant crowder volume density, but with increasing crowder radius (left to right).

Bound and unbound states in each binding free energy calculation were crowded separately. A box was created to contain both the protein complex (or each unbound state) and the model crowders, such that the box “walls” were each 70 Å from the most extreme (i.e., maximal and minimal) x, y, and z protein coordinates. The dimensions of the box were approximately 190×190×190 Å. Spherical crowders of either specified or random radii (up to 25 Å, roughly the size of the barnase–barstar complex) were added sequentially, and each potentially new crowder was accepted if it did not (1) overlap in space with any existing crowder or protein molecule, (2) partially or totally fall outside the total box volume, or (3) cause the volume density of crowders to be higher than the desired value. The volume density of crowders was calculated as the ratio of the total volume of the crowders to the originally available volume (i.e., volume not taken up by the protein(s)). Fig. 1 shows sample, random crowder placements around the bound state at denoted specifications. Preliminary analyses showed that one consequence of our crowder placement method is a depletion of crowder density at the system’s extreme edges; future efforts to place crowders could adopt a strategy leading to more even placement throughout the entire system volume.

### Continuum Electrostatics Calculations

A single-grid red-black successive over-relaxation finite-difference solver (M.D. Altman and B. Tidor, unpublished) [61] of the Poisson/Linearized Poisson Boltzmann Equation, distributed with the Integrated Continuum Electrostatics (ICE) software package (D.F. Green, E. Kangas, Z.S. Hendsch, and B. Tidor, Massachusetts Institute of Technology Technology Licensing Office), was used to solve for the electrostatic potentials of both crowded and uncrowded systems. Unless otherwise noted, a probe radius of 1.4 Å was used to define the molecular surface for the dielectric boundaries. Likewise, unless otherwise noted, a dielectric constant of 4 was used for all spherical crowders and protein atoms, and the solvent was modeled using a dielectric constant of 80. Potentials were solved on a 491×491×491 grid. A three-tiered focusing procedure was used, in which the system (the complex and all crowders) occupied 23%, 92%, and 184% of the grid. At the lowest focusing, the regions beyond the entire system were modeled as dielectric 80 and screened Coulombic (or Debye-Huckel, in cases of non-zero ionic strength) boundary conditions were used. Zero-radius dummy atoms were placed at identical extreme points of every run to maintain equal grid resolution for all states. At the highest focusing, this grid spacing yielded a resolution of approximately 4.6 grids/Å, and the grid was centered on barstar within the large system (for a small subset of runs, the grid was centered on a particular atom within the interfacial barstar Asp39 residue). PARSE radii and charges [62] were used. The ionic strength was set to zero except when implicit salt was modeled at a concentration of 0.145 M and a Stern layer of 2 Å was used. Due to memory limitations, runs with nonzero ionic strength were solved on a 401×401×401 grid, and to assess the effect of ionic strength, were compared only to other runs at the same grid resolution.

**Figure 2:**
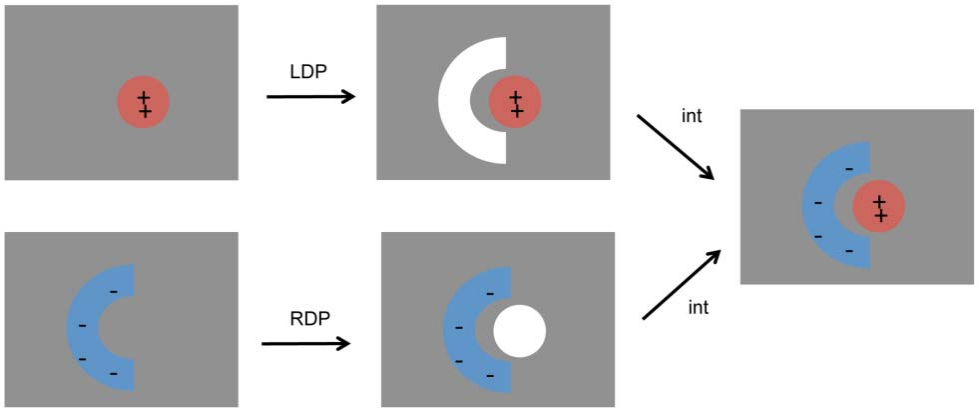
Schematic defining physically relevant components of the electrostatic binding free energy. Pictorially represented are the ligand (barstar) desolvation penalty (LDP), the receptor (barnase) desolvation penalty, (RDP) and the complex solvent-screened interaction (int). Gray regions denote solvent, and white regions denote low-dielectric cavities in the shape of a given partner, but without charges modeled. The total electrostatic binding free energy is LDP + RDP + int.

Potentials were solved for both the bound and unbound dielectric boundaries upon charging up one binding partner at a time. By multiplying (one-half) the potential differences due to charges on a given partner by the charges on that partner, desolvation penalties were obtained, and by multiplying the potentials due to charges on one partner by the charges on the other partner, solvent screened interactions were obtained [63] (Fig. 2).

### Model Charge Variation

The monopole on each binding partner was changed by adding or subtracting random charge values of maximum magnitude 0.1 e to randomly selected atoms within the partner until the desired overall monopole was reached. No single atom was allowed to have an overall charge magnitude greater than 0.85 e. To test the robustness of the results, monopoles were changed by starting both with the original charge distribution and from a structure in which all the charges were set to zero. Here we show only the results produced by starting with the original barnase-barstar charge distribution.

### Component Analyses

To quantify the contributions of selected residues toward the electrostatic component of binding in the presence and absence of model crowders, the partial atomic charges on the side chain of a given residue were all set to zero and the binding free energy re-evaluated, in a similar manner to component analyses in previous work on both protein and small molecule systems [55], [64]–[68]. The effect of zeroing out the side chain was then computed via:
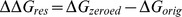
A positive value of **Δ**ΔG_res_ implies that a residue’s side chain contributes favorably toward the electrostatic component of binding, as zeroing out its charges worsens binding. The desolvation and interaction components of ΔΔG_res_ were computed by directly subtracting the desolvation and interaction components of the binding free energies between the system with zeroed charges and the original system, respectively.

### Component Analyses of Residue Groups within Barstar

For analyses in which charges of groups of residues were zeroed, groups were determined by calculating the solvent accessible surface area (SASA) of residues within each partner (assuming associated water molecules are considered residues and not bulk solvent) in the bound and unbound states. CHARMM was used to calculate SASA, using a 1.4 Å -radius probe and the CHARMM22 force field. Residues with non-zero burial upon binding were classified as either highly buried or peripheral depending on whether more or less than 50% of their unbound SASA remained in the bound state. Non-core residues were classified as either surface exposed or partially exposed depending on whether they have more or less than 50 Å^2^ SASA in the unbound state. Here, the charges of both side chain and backbone atoms were set to zero so that the union of all atoms considered was the entire barstar protein (and associated explicit water molecules).

### Data Analysis and Visualization

Figures of protein molecules and model crowder systems were generated using VMD [69]. All plots and data analyses were performed using Matlab (The Mathworks, Inc. Natick, MA).

## Results

To assess the effect of water depletion due to crowding on the electrostatic component of protein–protein binding, binding free energies were computed in the presence and absence of model crowders. To model the crowded states in a controlled fashion and focus on water depletion, spherical, uncharged “crowders” were randomly placed around the bound and unbound state proteins at specified densities (Fig. 1). The effect of crowding on the electrostatic component of the binding free energy was quantified as the difference between the electrostatic binding free energies in the presence and absence of crowders:
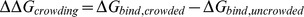
A negative ΔΔG_crowding_ means that crowding lowers the electrostatic binding free energy (i.e., favors binding, all other components equal). With our model, ΔG_bind,elec,uncrowded_ was found to be 0.5 kcal/mol, suggesting that the electrostatic component of binding in this system (in pure aqueous solvent) is neither strongly favorable nor unfavorable, in qualitative agreement with previous work using quantitatively different parameters [70]. Given that the electrostatic binding free energies between proteins are generally quite unfavorable with models using an internal dielectric constant of 4 [71], our value supports the accepted view that electrostatics play an important role in this system.

Binding free energy contributions were broken into desolvation and interaction components (Fig. 2). The free energy cost upon binding to remove solvent interactions with barstar (considered the “ligand”) is denoted the ligand desolvation penalty (LDP), and was found to be 41.7 kcal/mol for the uncrowded system. The energetic cost upon binding to remove solvent around barnase (the “receptor”) is termed the receptor desolvation penalty (RDP, 37.2 kcal/mol when uncrowded). Finally, the solvent-screened interaction between the partners (int) was also quantified (−78.4 kcal/mol when uncrowded).

### On Average, Crowding Lowers Desolvation Penalties and Amplifies Interactions

**Figure 3:**
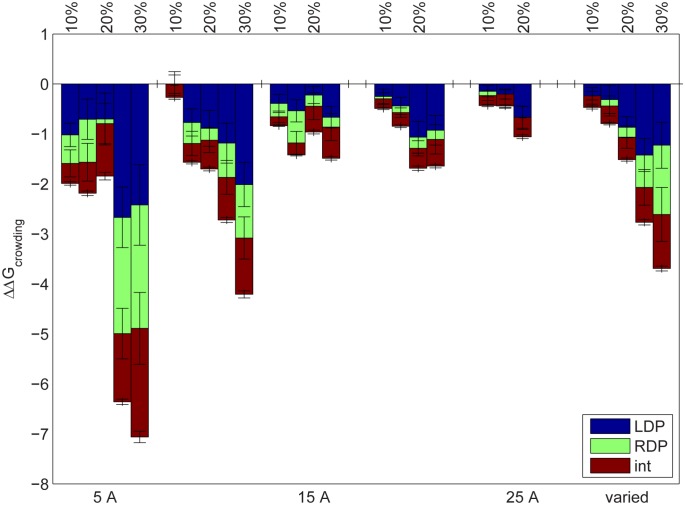
ΔΔG_crowding_, in kcal/mol, for barnase-barstar vs. crowder volume density (top axis) and radius (bottom axis). The bars at right (“varied”) are for systems in which the crowder radius varies within each trial. Each bar is the average of 50 trials and is shown as a composite of its contributions of barstar desolvation penalty (LDP, blue), barnase desolvation penalty (RDP, green), and solvent-screened interaction (int, red). Error bars on each contribution represent +/−1 standard error. Missing bars are a result of unsatisfiable geometric constraints (see Results).

Figure 3 is a graph of ΔΔG_crowding_ as a function of crowder radius (bars grouped by bottom axis) and crowder volume density (top axis). In the rightmost set of bars, crowder radii vary within each system from 5–25 Å (the largest spheres were therefore approximately the size of the protein complex). Total ΔΔG_crowding_ values are broken up into contributions due to changes in barstar’s desolvation penalty (LDP, blue), barnase’s desolvation penalty (RDP, green), and solvent-screened interaction (int, red). Each bar is the result of 50 random trials, with average values +/− standard error (not standard deviation) shown for each contribution.

Figure 3 shows that on average, ΔΔG_crowding_ was negative for all crowder densities and radii, although generally, the effects were more pronounced at higher crowder densities and smaller crowder radii. Moreover, the changes in all contributions (LDP, RDP, and int) were generally negative on average, in this system. This result makes intuitive sense – in a crowded environment, each unbound state is already partially desolvated by crowders, with some crowders potentially occupying the same space in the unbound state as the binding partner does in the bound state. Hence, there may be less solvent displaced near the binding interface upon binding in the crowded system when compared to an uncrowded one, resulting in a reduced desolvation penalty on average. Moreover, the bound state is also partially desolvated due to the crowding, resulting in less solvent screening and more amplified interactions between the two partners. Because the interactions in this complex are favorable in general, amplifying them would increase their favorability.

The average effects seen in Fig. 3 are qualitatively similar to what one might obtain using a lower solvent dielectric constant. Previous work has modeled aspects of crowding via the use of a lower “effective” solvent dielectric constant [37], [38], [46], [48], and experimental evidence suggests that a dielectric constant can be characterized for the cytoplasm [51], [53] through measuring shifts in emission wavelength maxima of fluorescent probes due to the polarity of the microenvironment. This observed constant likely is a macroscopic average accounting for both the loss of water mobility and water depletion (and potentially other effects), the first of which is not accounted for in the present study. Nevertheless, it is instructive to measure the effects of a lowered, effective solvent dielectric on protein–protein binding. Figure S1 shows ΔΔG values (relative to a solvent dielectric constant of 80) for the desolvation and interaction components of barnase-barstar binding as a function of solvent dielectric constant. In addition, Table 1 shows numerical data using two potential values of solvent dielectric constant – an experimentally obtained value of 21.9[53] and the value of 55, similar to values found from explicit simulations at 30% crowder volume density, to model solely the effects of hindered water mobility [47]. A dielectric constant of 21.9 produced ΔΔG values that were several times more pronounced (Table 1) than the results obtained using explicit crowders (Fig. 3), but this may be because the experimentally-obtained constant would account for not only water depletion, but also hindered water mobility and other possible effects of crowding. A dielectric constant of 55 again produced more pronounced results than using explicit crowders within a dielectric 80 medium, although the effects were more quantitatively similar to our explicit crowding simulations (∼1 kcal/mol difference in ΔΔG for desolvation components and ∼5 kcal/mol difference in ΔΔG for interaction, at a 30% crowding density and varied radius, Table 1). Again, differences could be due to the fact that this value was found to account for hindered water mobility and not water depletion.

**Table 1:** ΔΔG_crowding_ values for selected model systems described in the text.

ΔΔG_crowding_	LDP	RDP	int	TOT
*ϵ* **_out_ = 55**	−0.1	−1.1	−4.8	−6.0
*ϵ* **_out_ = 21.9**	−3.1	−6.6	−21.7	−31.4
*ϵ* **_in_ = 4, control run**	−1.2±0.5	−1.4±0.5	−1.08±0.05	−3.7±0.7
*ϵ* **_in_ = 1, same**	−3±2	−5±2	−1.4±0.1	−9±3
*ϵ* **_in_ = 1, random**	0±1	−5±1	−1.4±0.1	−6±2
**0 M ions, same, lower grid**	−1.2±0.5	−1.4±0.5	−1.08±0.05	−3.7±0.7
**0 M ions, random, lower grid**	−1.0±0.4	−0.1±0.3	−0.97±0.04	−2.1±0.5
**0.145 M ions, same, lower grid**	−0.9±0.5	−1.3±0.5	−0.48±0.05	−2.8±0.7
**0.145 M ions, random, lower grid**	−0.7±0.4	−0.2±0.3	−0.51±0.05	−1.4±0.5

ΔΔG_crowding_ values broken into components (LDP, RDP, int, and total) for systems not shown in Fig. 3. In the first two rows, the outer dielectric constant is varied as a substitute for explicitly modeling crowders. In the next set of rows (*ϵ*_in_ = 1, *ϵ*_in_ = 4), the internal dielectric constant was changed to 1 and compared with the control value of the reference system (*ϵ*_in_ = 4, also the rightmost bar in Fig. 3). The last four rows show the effect of nonzero ionic strength. For maximal control, all components were re-evaluated at a slightly lowered grid both without ions (“0 M ions, same, lower grid”) and with ions (“0.145 ions, same, lower grid”). Additionally, crowders were either kept the same as they were in the 50 trials of the reference system (“same”) or were randomly varied (“random”).

The qualitative trends seen with lowered dielectric constants (Fig. S1) were similar to the trends found in this work for either increasing crowder volume density or decreasing radius, although for a given crowder radius and volume density, there may not exist an effective dielectric constant that provides quantitative agreement. Perhaps a “long-range” dielectric constant cannot model the full effect of hydration immediately surrounding each macromolecule; in a heterogeneous environment, the dampening of the electric fields due to a small amount of highly polar water might not be captured by an average, low macroscopic dielectric constant and therefore, effects of crowding may be overestimated. Nevertheless, one potential solution, similar to what was done in work by Harada *et al.* [38], is to use a slightly lower dielectric constant to account for the loss of water mobility and explicitly model crowders to account for water depletion. Future work could also involve effective medium theory approaches to estimate effective dielectric constants of this composite environment as a function of crowder size and shape [72].

The relatively small standard error for interaction indicates that the mean stabilization due to the further descreening of interactions relative to infinite dilution is fairly robust to the ensemble of states sampled; there is little uncertainty in the mean effect. However, the large standard error for both desolvation contributions in all ensembles indicates great uncertainty in the mean reduction of desolvation penalties due to random crowder placement. As desolvation penalties depend strongly on the level of direct solvent exposure of charged or polar interfacial groups, it makes sense that they will be very sensitive to precise crowder placement. Interaction energies, on the other hand, are more long-ranged, except for interfacial interactions (and these are fairly unaffected by crowders in the bound state anyhow), and are therefore far less sensitive. The large standard error due to desolvation, by definition, implies an even larger standard deviation and therefore a huge amount of variability between trials, which suggests the necessity of thorough sampling. Currently, it is computationally infeasible to thoroughly sample all relevant crowder configurations. Preliminary attempts to use Boltzmann-weighting to more heavily account for lower-energy states by obtaining partition functions from each set of 50 sampled configurations resulted in similar qualitative trends to those shown in Fig. 3 (data not shown).

Our results suggest that the effects of crowding on water depletion are most pronounced at a given crowder volume density when the crowders are small, although large standard errors confound the robustness of this result, especially for desolvation. Presumably, very small molecules can more closely approach the irregular surface of a protein, more substantially desolvating it in its unbound state and more effectively descreening its interactions with a partner in the bound state relative to infinite dilution. Analyses of our model crowded systems showed that the minimum distance of approach between any one crowder and the proteins increases on average as the crowder radius increases (Figure S2), in support of this hypothesis.

It is plausible that aspects of this observed trend could be dependent on the use of a standard, nonzero-sized (here, 1.4 Å) “probe” used to generate the molecular surface in continuum models. The water-sized probe is intended (as standard practice) to approximately account for the nonzero size of discrete water molecules and the inability of “actual” water molecules to occupy cavities and crevices smaller than their size. A consequence of this model feature is that low-dielectric regions will be larger than the actual volume occupied by model crowders and protein, and this difference will likely be greater for systems with smaller-radius crowders due to the likelihood that they often closely approach each other and the protein.

To test this hypothesis, we redid a subset of the calculations shown in Figure 3 using a probe radius of zero to generate the molecular surface. The results are shown in Figure S3. Desolvation penalties were still reduced on average and interactions amplified, but as expected, the quantitative effects were now often ∼50–75% less pronounced (ΔΔG_crowding_  =  ∼2 kcal/mol or less). Additionally, the dependence of the desolvation effects on radius was not apparent (although they did not appear to be statistically significant even with a standard probe radius). However, the average effect on the interaction component still strengthened overall as the crowder radius decreased, suggesting some robustness to the observation that smaller crowders may have greater impact. While it is standard practice to use a probe radius of 1.4 Å [73], [74], results using a continuum model can be sensitive to this feature [74], [75]. Our results demonstrate this limitation, specifically when modeling crowding effects using a continuum approach.

Even with the “standard” probe radius of 1.4 Å, at radii that more accurately model small proteins (20–25 Å), the mean effects on electrostatic interaction were found to be modest, but still significant on average, especially at higher crowding densities. These data suggest that the effects of crowding on electrostatics could be sensitive to the precise distribution of molecular sizes within the cell, and that it might be not be crowding due to proteins but rather, due to smaller metabolites and peptides that most greatly affects the electrostatic component of binding. We note that the trends for radii are curtailed here due to missing data at higher crowder densities and larger radii. Because of our purely random, sequential crowder placement, it became geometrically impossible to satisfy all constraints noted in the Methods when both crowder size and desired volume density were large. Future work can attempt to explore this region of property space while still maintaining a purely random crowder placement within the noted constraints.

Taken together, these results show that on average, the effects of crowding on electrostatic interactions can vary as a function of both crowder volume density and size, but desolvation effects are highly sensitive to crowder placement. To qualitatively account for crowding effects due to water depletion, therefore, it may be expedient to use an effective lowered solvent dielectric constant. Our work supports the idea that such a constant is likely to be specific to crowding volume fraction [47] and the distribution of crowder radii, and additional parameters may be needed to capture system-specific variations due to various arrangements of crowders.

In addition to the varied probe radius size discussed above, a subset of data was obtained under other different model conditions, to gauge the robustness of our results to parameters and physical conditions. First, we varied the internal dielectric constant used for both protein and model crowders. For maximal control, the precise locations of crowders in the bound and unbound states of the 50 trials were maintained in calculations with different dielectric constants in one set of runs, and allowed to vary in another set. Results here used a varied crowder radius at a volume density of 30%. With an internal dielectric constant of 1, results were qualitatively similar to those with an internal dielectric constant of 4 when controlling for crowder placement and quantitatively more pronounced on average, especially for desolvation penalties (Table 1, *ϵ*_in_ = 1, same”). However, standard errors were much larger, which may explain the difference in ΔΔLDP_crowding_ between trials in which the same crowders were used and when random crowders were used (Table 1, “*ϵ*_in_ = 1, random”).

To understand how the presence of electrolytes could modulate the effect of crowding, data were gathered including implicit mobile ions at a concentration of 0.145 M through obtaining potentials via the linearized Poisson-Boltzmann equation. Again, we used a crowder volume density of 30% and randomly varied crowder radii, although all relevant runs with and without mobile ions were done at a somewhat lower grid resolution due to memory limitations when modeling salt (see Methods). We obtained qualitatively similar results when the solvent contained implicit, mobile ions, although the average lowering of the LDP, RDP, and especially int, were not as pronounced (Table 1).

**Figure 4:**
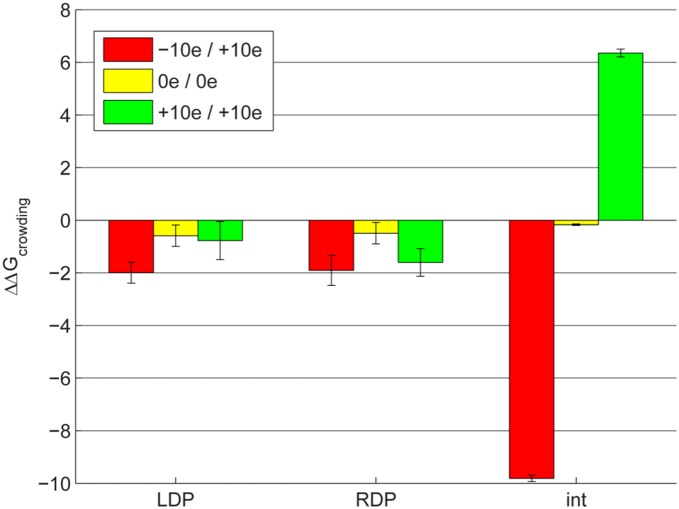
Effect of partner monopole on ΔΔG_crowding._ ΔΔG_crowding_, broken into barstar desolvation penalty (LDP), barnase desolvation penalty (RDP), and solvent-screened interaction (int) components, is shown in kcal/mol for the binding free energy of hypothetical proteins generated by randomly altering the charges of randomly selected atoms on the barnase–barstar complex until a desired overall monopole on each partner is reached (see legend). Each bar shows the average of 50 trials in which the bound and unbound states were crowded with spheres of random, varied radii (5–25 Å) to 30% crowder volume density. Error bars indicate +/−1 standard error.

If crowders descreen interactions relative to infinite dilution, they should amplify both attractive and repulsive interactions. To show this, we computationally modified the charge distributions on both barstar and barnase to vary their monopoles (see Methods). Of course, such charge distributions are not realistic, but they allow for a controlled, systematic study on how a system’s charge distribution may affect its molecular recognition profile in a crowded environment. Figure 4 shows the average change in LDP, RDP, and int for three modeled pairs of monopoles – in which the partners either had opposite, large-magnitude monopoles (+/−10 e), no net monopole, or the same, large-magnitude monopole (+10 e). Each bar is the average of 50 trials in which crowders of varied (5–25 Å) radius were used at a 30% volume density. The average effect of crowding on desolvation penalties was similarly stabilizing in all three cases, but the average effect on interactions is markedly different in the three cases. As expected, crowding greatly destabilized the (+10/+10) interaction and greatly stabilized the (+10/−10) one. This suggests that binding partners’ overall monopoles can affect how they interact with partners in a crowded environment, although this effect is mediated more by interactions rather than the desolvation component.

### Crowding can Differentially Affect Electrostatic Contributions of Side Chains toward Binding

Many protein–protein interactions have been shown to be mediated by one or more polar or charged residues or “hot-spots” [76]–[79]; such residues can be elucidated by experimental mutagenesis studies (e.g., alanine scanning) or through computational analyses. Presumably, if the overall electrostatic binding free energy can be modulated by the level of environmental crowding, as the model above suggests, then this implies that the specific contributions of individual residues toward that interaction can also be altered, but the nature of the alteration may depend on the properties of each residue.

To explicitly demonstrate, quantify, and better understand this intuitive idea, we began with the original (unaltered) charge distribution of the complex and quantified the electrostatic contribution of selected barstar residues toward the binding free energy by computationally setting the original partial atomic charges on a given side chain to zero and re-evaluating the binding free energy to obtain a ΔΔG_res_ (see Methods); this procedure was done both in the presence of crowders (the 50 trials used in the original analyses were used to obtain an average ΔΔG_res_) and in the absence of crowders. Consequently, we can define a ΔΔΔG_res,crowding_ that quantifies the effect of crowding on a residue’s contribution toward the binding free energy:
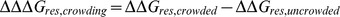
A positive ΔΔΔG_res,crowding_ means that a residue contributes *more* favorably (or less unfavorably) toward binding in the presence of crowding than in its absence.

**Figure 5:**
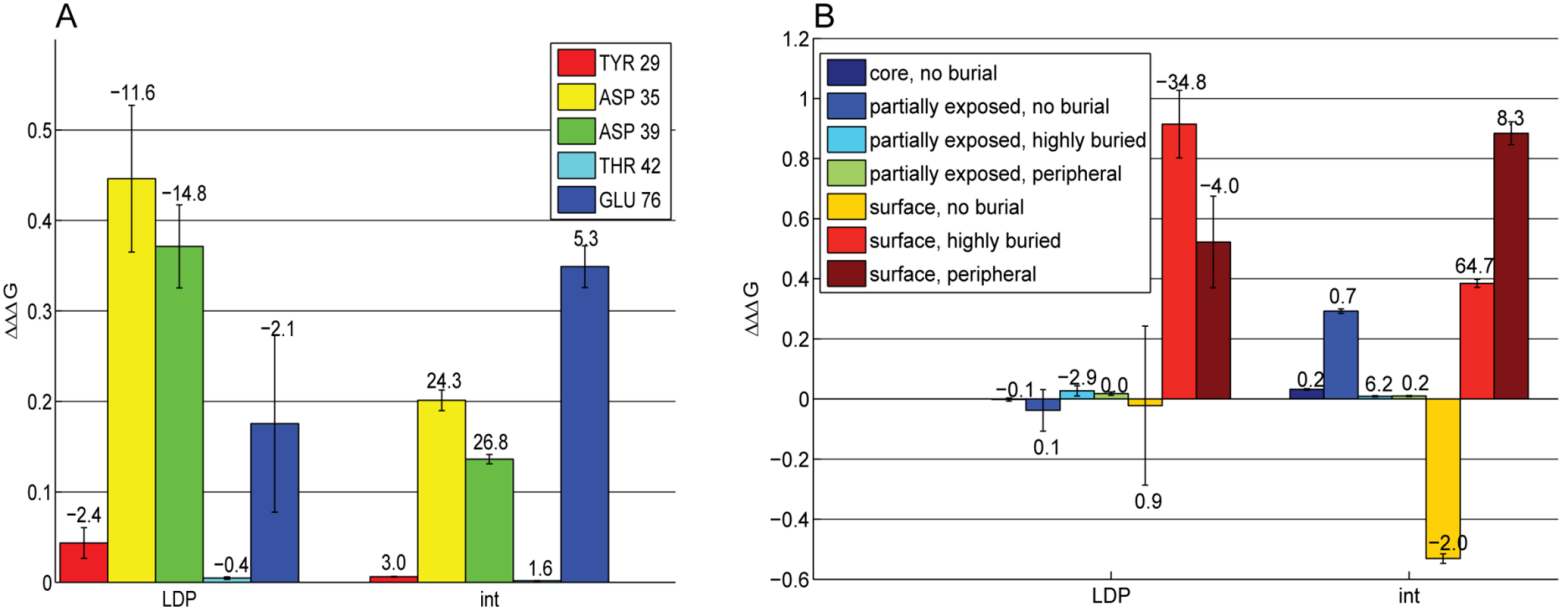
Effect of crowding on residue-based electrostatic contributions. ΔΔΔG_res,crowding_, broken into barstar desolvation penalty (LDP) and interaction (int), in kcal/mol, is shown for (a) selected barstar residues (see legend) and for (b) groups of barstar residues based on level of surface exposure and degree of burial (see Methods); The number above each bar indicates the actual magnitude of the selected component of ΔΔG_res_ without crowding present. Each bar indicates an average of 50 trials in which each crowded bound and unbound state is crowded with spheres of random, varied radii between 5 and 25 Å to 30% crowder volume density. Error bars indicate +/−1 standard error.

In this study, we chose to calculate ΔΔΔG_res,crowding_ for five barstar residues whose side chains were previously shown to contribute significantly toward the electrostatic component of binding free energy [55]: Tyr29, Asp35, Asp39, Thr42, and Glu76. Figure 5a is a graph of the ΔΔΔG_res,crowding_ for each of these residues, broken up into barstar desolvation (LDP) and interaction (int) components (there is no change in the desolvation of barnase, RDP, as only charges on barstar were changed to zero). On average, the charged side chains contributed even more favorably in the presence of crowding, although the effect was quite small, with an average ΔΔΔG_res,crowding_ of only tenths of a kcal/mol. The contributions were not significantly changed on average for the two polar side chains studied.

**Figure 6:**
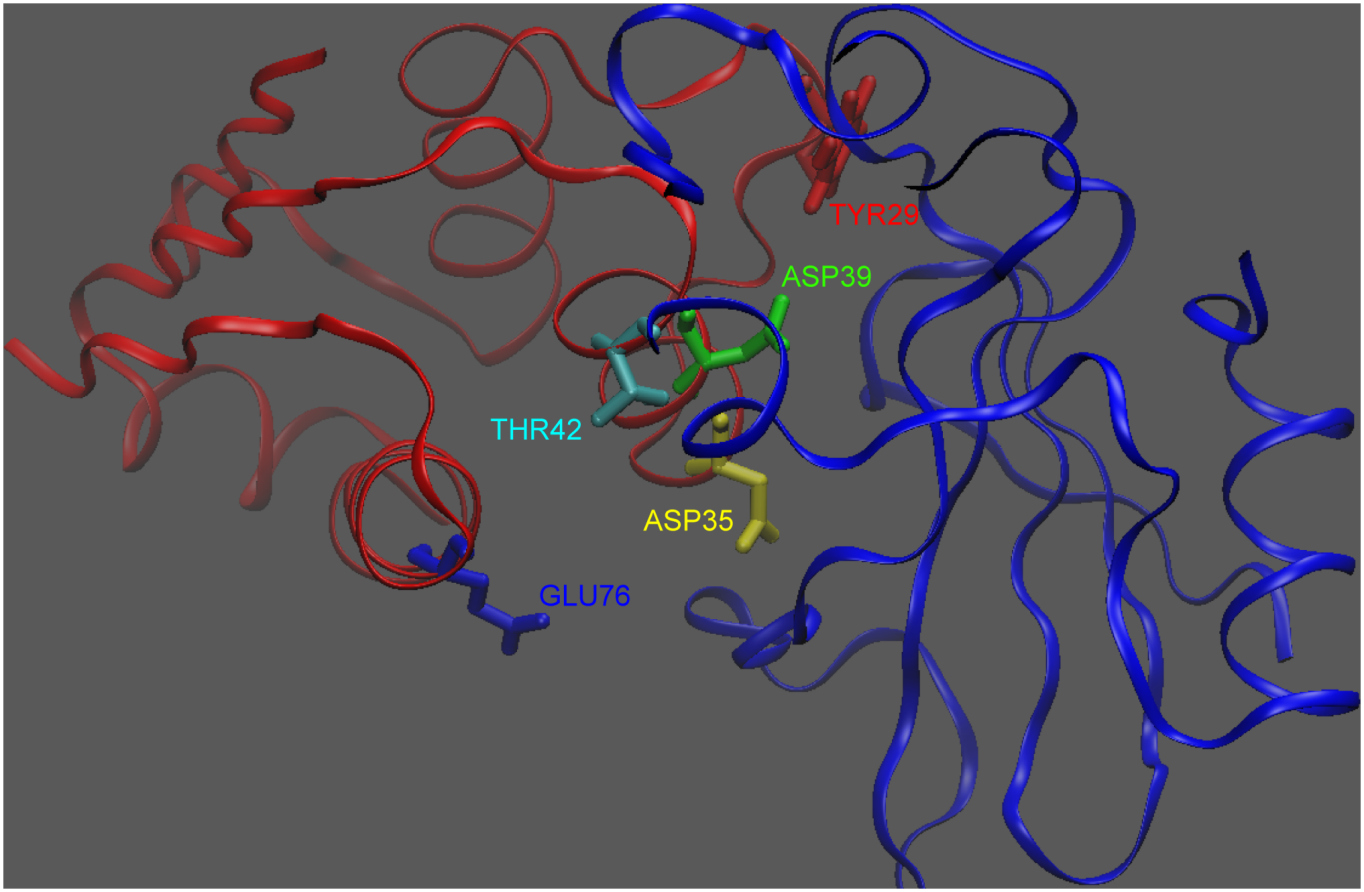
Location of the 5 barstar residues studied via component analysis within the barnase(blue)/barstar(red) complex.

Interestingly, the desolvation component of ΔΔΔG_res,crowding_ was altered more on average for Asp35 and Asp39, whereas the interaction component was altered more on average for Glu76. We hypothesize that the different mechanisms of altering ΔΔΔG_res,crowding_ is due to where these residues lie relative to the binding interface (Fig. 6). Both Asp35 and Asp39 are interfacial and highly buried upon binding, and so crowding may more greatly affect their desolvation penalties, by partially desolvating them already in the unbound state. Glu76, however, is more peripheral to the interface and so it remains more solvent exposed upon binding–this implies that crowding could more greatly impact the solvent-screening of its interactions in the bound state.

To further explore the idea that crowding might affect residue-based contributions differently, we grouped barstar residues based on both level of surface exposure and degree of burial upon binding (see Methods). Then, we zeroed out the charges simultaneously on all residues in each group (including both side chain and backbone) to determine ΔΔG_res_ for that group. This was done both in the presence and absence of crowding to obtain a ΔΔΔG_res,crowding_ (using the 50 trials used in the original analyses). Indeed, surface residues that are highly buried upon binding showed the largest desolvation component of ΔΔΔG_res,crowding_ values (Fig. 5b), while surface residues that are peripheral to the interface (i.e., only partially buried upon binding) showed the largest interaction component of ΔΔΔG_res, crowding_. Interestingly, ΔΔΔG_res,crowding_ of surface residues with no burial upon binding (i.e., distal from the interface) was negative; here, crowding makes these residues contribute *more unfavorably* toward binding. This result may be due to the dominating effect of the monopoles of distal groups; the monopoles on our model of barnase (+1) and the collection of distal, surface exposed residues on barstar (+3) have the same sign. The same trends are found when one controls for the number of residues in each group by finding the average ΔΔΔG_res,crowding_ per residue in each group (Fig. S4). These results explicitly demonstrate that electrostatic contributions–and therefore perhaps mutational energies–can be predictably altered in an environmentally-dependent way for residues in a crowded environment.

## Discussion

In this work, we used simplified models to investigate the effect of macromolecular crowding on the electrostatic component of protein–protein binding free energy via water depletion. We found that for proteins with favorable electrostatic interactions, crowding can enhance the relative favoring of the bound state due to lowered desolvation penalties and enhanced interactions. For proteins with potentially unfavorable interactions, there may be opposing effects. The effects of crowding on desolvation were highly sensitive to crowder placement–yielding far more uncertainty in the mean effect on desolvation than in the mean effect on the interaction component.

Our results can potentially provide experimentally-testable hypotheses. For example, one could experimentally study the effect of monopole-changing yet relatively isosteric (e.g., Asn→Asp) interfacial and peripheral mutations on protein–protein binding in crowded and uncrowded environments to see if crowding affects their relative contributions as predicted.; these experiments can be bolstered by varying ionic strength to highlight the interaction component of binding over desolvation components. Experimental tests would likely combine the effects of crowding due to both water depletion and lowered solvent mobility, so experimental results should reflect the predictions in this work in combination with other computational predictions [47].

The importance of crowder size was studied in a previous computational study that focused on the excluded volume effect of crowding on the binding of the barnase–barstar complex [35]. Like our study, it was also found that smaller crowders had a larger effect, but for a different reason–at a given volume density, smaller crowders left smaller voids for the proteins to occupy, lowering the available volume. This effect was confirmed in another study, and it was also shown that the ratio between crowder size and protein size is important [12]. Thus, smaller crowders may have a bigger impact for multiple reasons – by their excluding more volume and by their ability to more closely approach proteins to desolvate them and descreen their electrostatic interactions relative to infinite dilution.

We also demonstrated that crowding can differentially affect the relative contributions of residues toward binding. That these changes can be dominated by different phenomena (desolvation vs. interaction) could provide avenues for rational, environmentally-dependent design tasks.

This study provides a useful framework on which to build in future studies. With adequate computational resources, larger-sized model crowders and overall crowded volumes could be explored. Elements of “reality” can be added individually, in turn, to understand the effect of each on the binding free energy. Such elements include using actual protein shapes for the crowders (crowder shape has been shown to affect changes in folding and binding free energies [12], [80]) as well as protein charge distributions to include direct enthalpic crowder interactions, which have been shown to be important for protein stability and conformation [18], [48]; it would be interesting to quantify their precise effects on protein–protein binding. Another future goal is to increase the sampling of crowder configurations and potentially the conformational states of the binding partners, to allow for Boltzmann-weighted averages through Monte Carlo or dynamic simulations. In this study, the costs of Poisson-based models on such large systems prohibited exhaustive sampling (each binding free energy calculation took ∼0.5 day of CPU time and>1GB RAM with current resources).

To also account for the altered mobility of water molecules due to crowding, explicit solvent simulations are necessary, and have been previously attempted [38], [47], although rigorously analyzing such effects on the energetics of specific protein–protein binding has yet to be done, to our knowledge. Given the potential computational cost of such studies, alchemical transitions [81], [82] of individual residues (i.e., component analysis) or small molecule–protein binding systems may be good starting points.

In this study, we demonstrated and systematically explored the idea that macromolecular crowding can affect the electrostatic component of the free energy of binding between proteins through depleting regions of high dielectric water. Our results highlight yet another example of how environmental effects can have a quantitative and potentially qualitative impact on molecular recognition and should therefore be considered in both the analysis and the rational design of biomolecular systems.

## Supporting Information

Figure S1 **ΔΔG_elec_ vs. solvent dielectric (relative to a solvent dielectric constant of 80), without explicit crowders.** A lowering of the external dielectric constant produces a similar qualitative trend as increasing the volume density or decreasing the radius of explicit crowders.
(PDF)

Figure S2 **Average minimum distance of approach between crowders and protein vs. crowder radius.** The minimum distance of approach is the shortest distance between the protein and crowder in each state, accounting for their radii. Data are shown for both 15% crowder volume density (data for 20% crowder density show a similar trend, not shown). Data are averaged over bound and unbound states for all 50 trials conducted for each radius and volume density. Error bars are +/− one standard deviation.
(PDF)

Figure S3 **Effect on ΔΔG_crowding_ of using a zero-radius probe to generate the molecular surface.** A subset of runs shown in Fig. 3 were redone using a zero-radius probe sphere to generate the molecular surface instead of the standard 1.4-Å probe. Identical crowder placements were used for each bar shown here and the bar corresponding to the same crowder density and radii in Fig. 3; the only different is in the size of the probe sphere.
(PDF)

Figure S4 **Per residue ΔΔΔG for sets of residues on barstar.** Residues were grouped by degree of burial and solvent exposure and values were *normalized by dividing by the number of residues in each group* (Figure 5b in the main text does not normalize per residue). Similar overall qualitative trends are seen in this Figure and in Figure 5b in the main text. The number above each bar indicates the per-residue value of the selected component of ΔΔG_res._
(PDF)

File S1 **Contains sample pdb files used in our analyses; each contains the barnase/barstar complex with a specific random placement of crowders of varying radii at 30% crowding density.** Also included are files specifying, for each pdb file, the radii of each atom and model crowder.
(TAR)
