# Supplementary figures and images for: The Effect of Macromolecular Crowding on the Electrostatic Component of Barnase–Barstar Binding: A Computational, Implicit Solvent-Based Study
Source: PLoS One. 2014 Jun 10;9(6):e98618. doi: 10.1371/journal.pone.0098618 (PMC4051634; doi:10.1371/journal.pone.0098618)

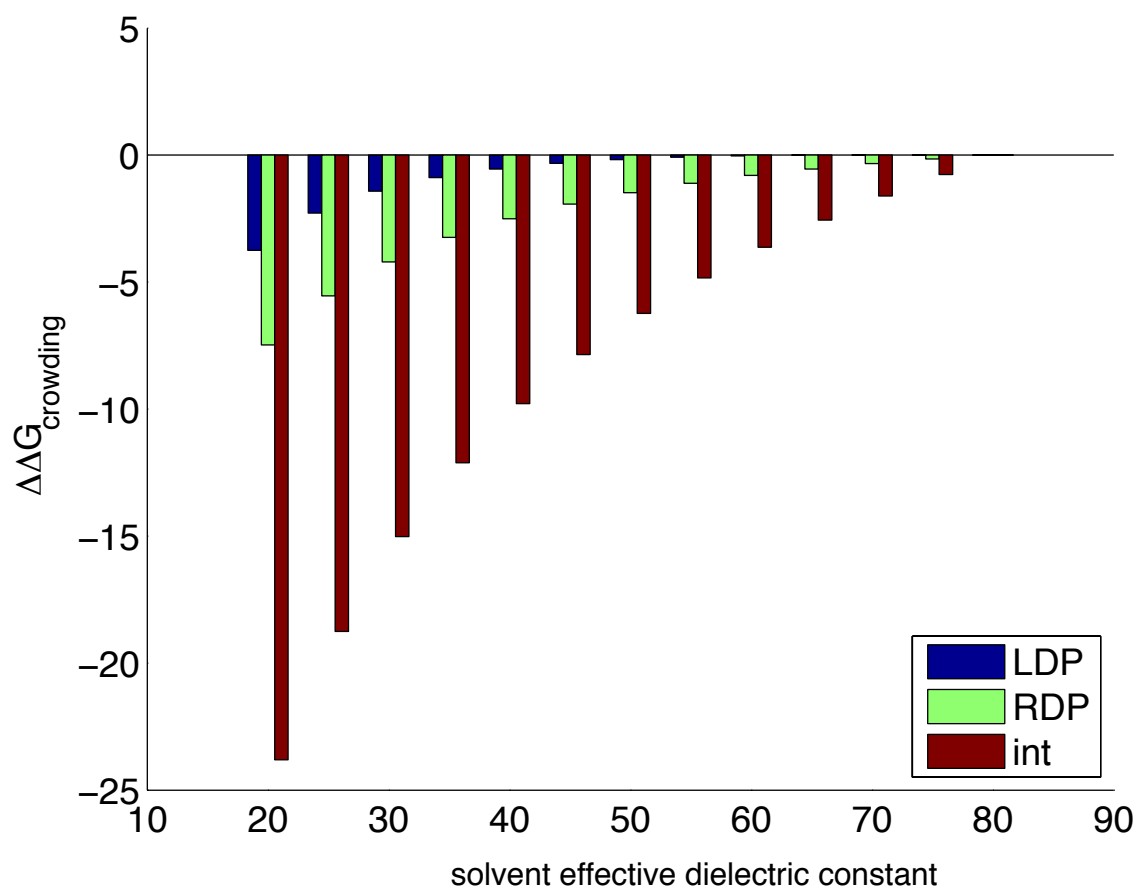

Supplement: Figure S1 — ΔΔGelec vs. solvent dielectric (relative to a solvent dielectric constant of 80), without explicit crowders. A lowering of the external dielectric constant produces a similar qualitative trend as increasing the volume density or decreasing the radius of explicit crowders. (PDF) [file pone.0098618.s001.pdf]

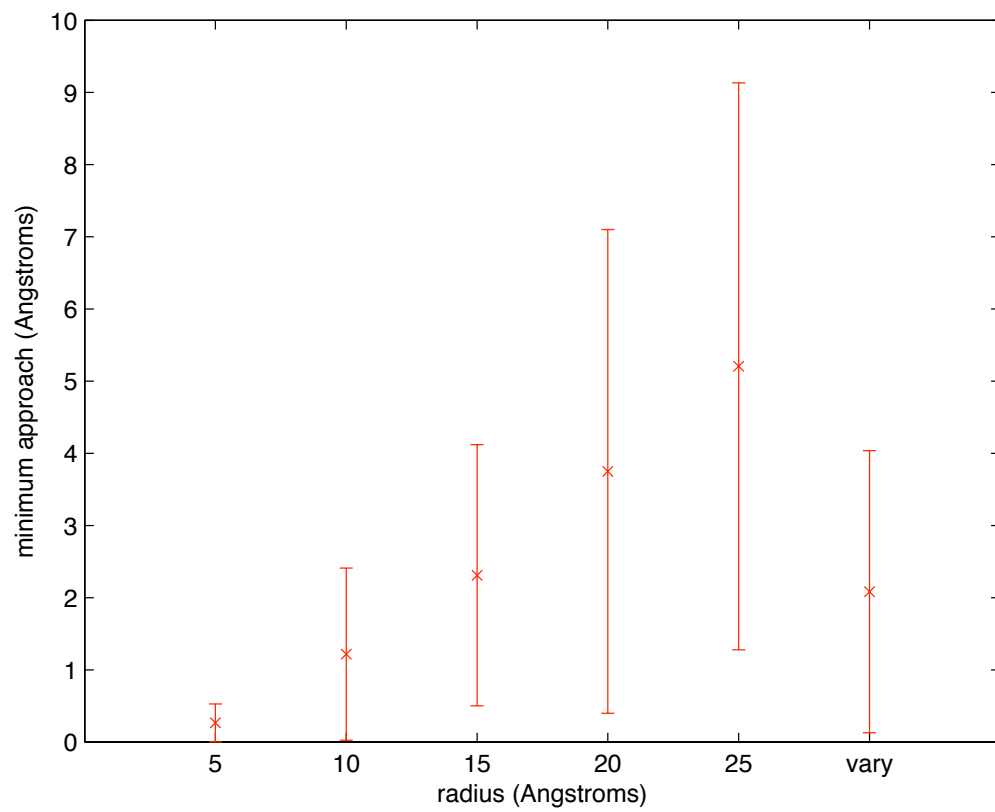

Supplement: Figure S2 — Average minimum distance of approach between crowders and protein vs. crowder radius. The minimum distance of approach is the shortest distance between the protein and crowder in each state, accounting for their radii. Data are shown for both 15% crowder volume density (data for 20% crowder density show a similar trend, not shown). Data are averaged over bound and unbound states for all 50 trials conducted for each radius and volume density. Error bars are +/− one standard deviation. (PDF) [file pone.0098618.s002.pdf]

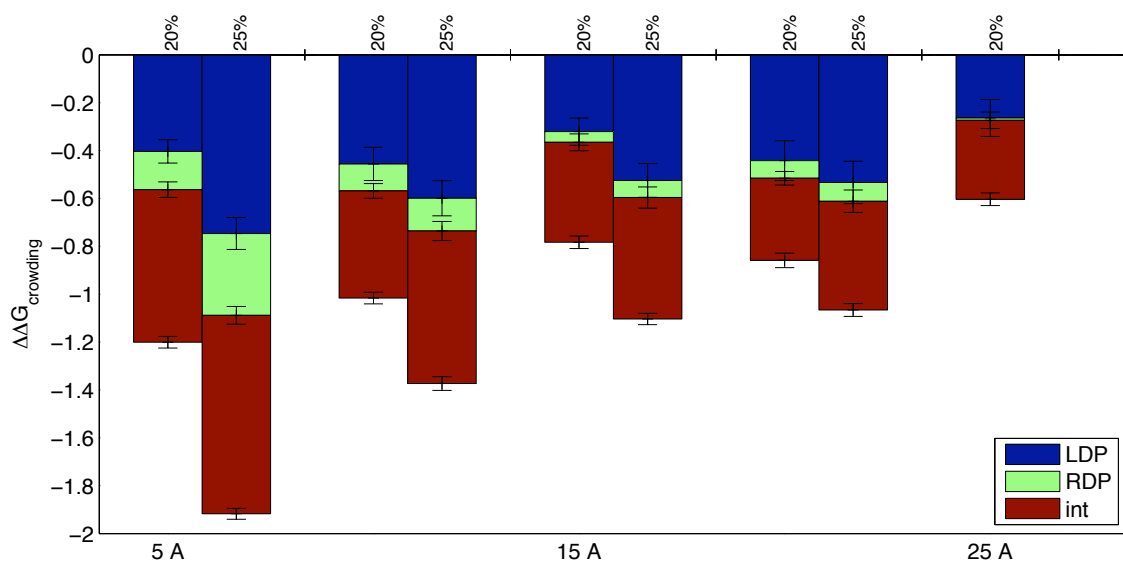

Supplement: Figure S3 — Effect on ΔΔGcrowding of using a zero-radius probe to generate the molecular surface. A subset of runs shown in Fig. 3 were redone using a zero-radius probe sphere to generate the molecular surface instead of the standard 1.4-Å probe. Identical crowder placements were used for each bar shown here and the bar corresponding to the same crowder density and radii in Fig. 3; the only different is in the size of the probe sphere. (PDF) [file pone.0098618.s003.pdf]

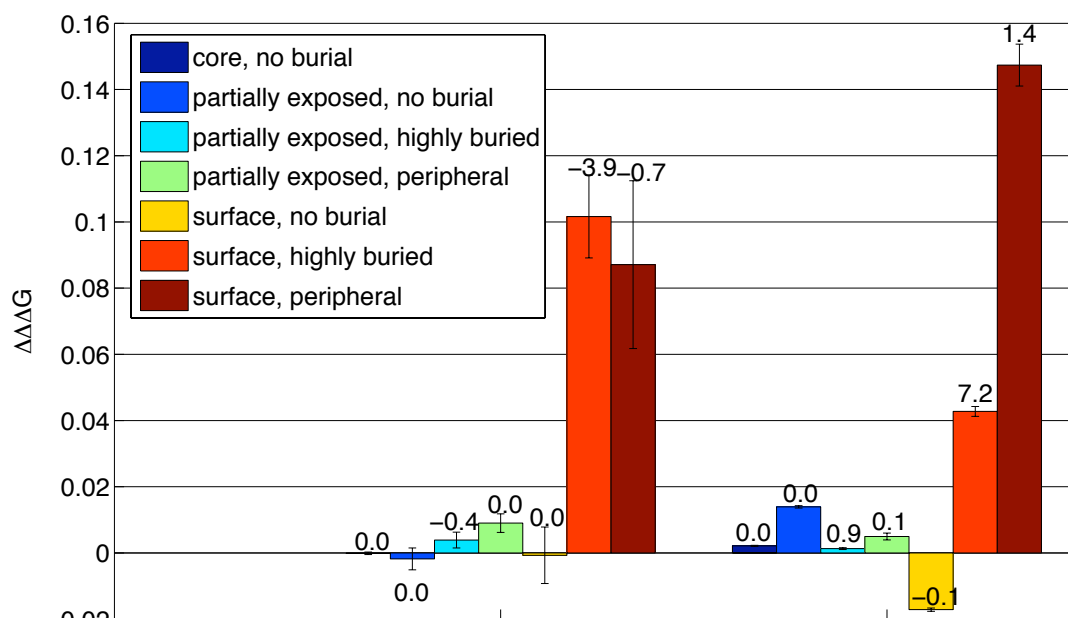

Supplement: Figure S4 — Per residue ΔΔΔG for sets of residues on barstar. Residues were grouped by degree of burial and solvent exposure and values were normalized by dividing by the number of residues in each group (Figure 5b in the main text does not normalize per residue). Similar overall qualitative trends are seen in this Figure and in Figure 5b in the main text. The number above each bar indicates the per-residue value of the selected component of ΔΔGres. (PDF) [file pone.0098618.s004.pdf]
